# From overlooked to just right: putting the focus on metronidazole infusion timing for colorectal surgical prophylaxis

**DOI:** 10.1017/ash.2025.10260

**Published:** 2026-01-02

**Authors:** Peter Oakes, Lindsay E. Donohue, Sunny Chiao, Susan Ketcham, Traci Hedrick, Rachele Olivier, Amy J. Mathers

**Affiliations:** 1 Division of Infectious Diseases & International Health, Department of Medicine, University of Virginia Health, Charlottesville, VA, USA; 2 Department of Pharmacy, University of Virginia Health System, Charlottesville, VA, USA; 3 Department of Anesthesiology, University of Virginia Health System, Charlottesville, VA, USA; 4 Perioperative Services, University of Virginia Health System, Charlottesville, VA, USA; 5 Colon and Rectal Surgery, Division of General Surgery, University of Virginia Health System, Charlottesville, VA, USA; 6 Department of Quality and Performance Improvement, University of Virginia Health System, Charlottesville, VA, USA

## Abstract

Preoperative prophylaxis efficacy depends on antibiotic infusion completion before incision. In our colorectal program, metronidazole often wasn’t completed on time, in contrast to cefazolin. A multidisciplinary effort standardized its administration timing, improving infusion completion rates. We evaluated the impact of this intervention on surgical site infections in colorectal surgery.

## Introduction

Both the timing and selection of antimicrobial prophylaxis (AP) reduce the risk of surgical site infections (SSI), but the logistics around varied infusion times are often overlooked.^1^ National AP guidelines jointly issued in 2013 by the American Society of Health-System Pharmacists (ASHP), the Infectious Diseases Society of America, the Surgical Infection Society, and the Society for Healthcare Epidemiology of America recommend that AP should be infused (both initiated and completed) within a 60 min window before incision.^2^ However, this recommendation is based primarily on studies focused on first- and second-generation cephalosporins infused via rapid intravenous (IV) push over a number of minutes by an anesthesiologist prior to skin incision. Vancomycin and fluoroquinolones are well known exceptions and are permitted a longer 120 min infusion window, due to rate limitations precluding the ability to completely administer doses in the shorter time frame. Subsequent studies have validated the consequences of failing to adhere to this earlier window for vancomycin, noting increased SSI rates when infusions are initiated too close to the time of incision and describing the significant resource allocation required to coordinate the complex logistics of an operating room setting.^3,4^ Despite the need for slow IV drip administration and a long half-life with active metabolites promoting adequate tissue concentrations for prolonged periods of time, metronidazole (MTZ) has not been clearly specified in this guideline carve-out, and one prior study has noted challenges with completing MTZ infusions prior to skin incision when given as a 30 min infusion by anesthesia in the operating room.^5^

MTZ IV infusion is only commercially supplied as a diluted and neutralized 500 mg/100 mL solution, as the undiluted reconstituted product is not appropriate for direct IV injection due to its low pH. In the United States, it is indicated for use as preoperative AP in patients undergoing elective colorectal surgery (COLO) at a dose of 15 mg/kg (approximately 1 g for a 70 kg patient) infused over 30 – 60 min and completed approximately 1 hour prior to incision, followed by intraoperative re-dosing every 6 hours with 7.5 mg/kg (i.e., ∼500 mg).^6^ Alternatively, the 2013 ASHP guidelines suggest an initial MTZ dose of 500 mg preoperatively within 60 min without any intraoperative re-dosing.^2^ While institutional practices vary, published evidence to support the safety of infusions over less than 15 – 30 min is sparse.^7^

Although other AP options exist that provide the spectrum of activity required for procedures entering the gastrointestinal tract, the combination of cefazolin and MTZ is preferred by our institution, a 600-bed academic medical center, given studies suggesting lower rates of SSI when compared to cephamycins^6^ and the unnecessary additional spectrum of ertapenem. Longitudinal quality and performance improvement efforts around COLO SSI at our institution are coordinated via a multidisciplinary coalition comprising key stakeholders in Hospital Epidemiology and Infection Prevention, Colorectal Surgery, Perioperative Nursing, Anesthesiology, Antimicrobial Stewardship, and Pharmacy. As part of our investigation into opportunities for improvement with respect to AP, we noted that MTZ initiation was frequently documented to be given by anesthesia within just minutes of, or even after, incision in the operating room. Herein, we report the results of a focused intervention to optimize timing of preoperative COLO AP, with a focus on MTZ.

## Methods

**Figure 1.**
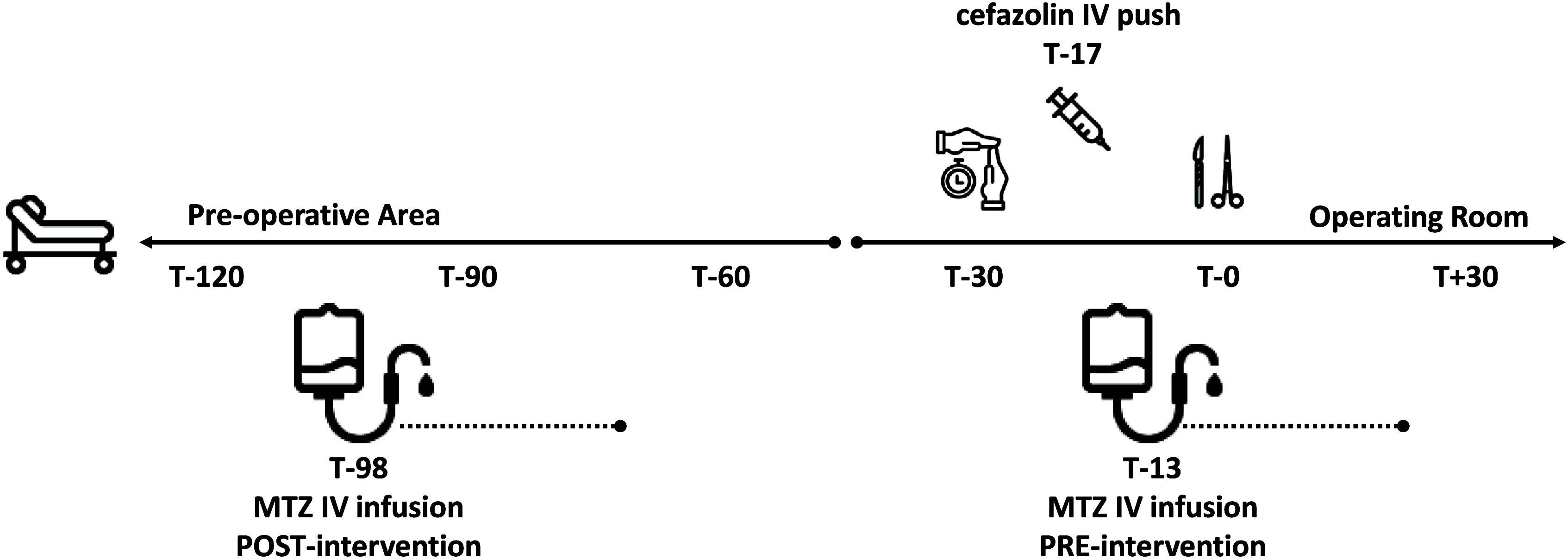
Schematic detailing change in metronidazole infusion timing and location. (T-0, time of incision; IV, intravenous; MTZ, metronidazole).

We postulated that we could improve the number of COLO cases receiving a complete MTZ infusion within an expanded goal time frame of 120 min preincision by shifting administration to a preoperative nursing responsibility (Figure 1). To support this, we updated our electronic medical record (EMR; EPIC, Madison, WI) preoperative COLO order set to include the following MTZ administration instructions: “To be given by preop nurse via infusion pump over 30 min within 120 min PRIOR TO procedure.” Education was provided to all involved services affected by this change, including surgeons, pharmacists, preoperative nursing staff, and anesthesia providers. Following implementation, we compared baseline characteristics, urgency class, rates of correct AP infusion, and incidence of SSI among all patients undergoing COLO procedures in the pre- (6/19/2022 – 10/18/2023) and postintervention (10/19/23 – 2/19/2025) periods using data extracted via custom query of our EMR reporting database supplemented by paired chart review.

## Results

Patients in the preintervention group were younger and more likely to undergo an elective procedure (Table 1). In the preintervention group, MTZ doses were seldom initiated and completed within the specified preincision window of 60 min (< 1%, *n* = 4/671) with a median initiation time of only 13 min, too close to incision to allow for complete infusion (i.e., < 30 mins) (Table 1). Shifting the infusion to preoperative nursing and widening the allowable preincision initiation window significantly improved the proportion of doses at goal postintervention (49%, *n* = 332/671), with a median MTZ initiation time of 98 min prior to incision. In contrast, there was no change in the proportion of correctly infused cefazolin doses. There was not a significant difference in the incidence of SSI (from 46.5 per 100,000 cases to 50.9 per 100,000 cases).

**Table 1.** Baseline demographics and outcomes

Characteristic	Preintervention N = 754	Postintervention N = 747	*P*-value
**Baseline demographics**			
**Mean age (years)**	58	60	< .001
**Sex assigned at birth: female**	382 (51)	363 (49)	.44
**Race: white/Caucasian**	631 (84)	624 (84)	.94
**Mean body mass index**	28	28	.7
**Diagnosis: Diabetes mellitus**	129 (17)	126 (17)	.89
**Case urgency classification**			
**Elective (scheduled based on available OR time)**	626 (83)	575 (77)	.004
**Emergent (to OR within 1 h)**	50 (7)	48 (6)	.92
**Priority (to OR within 2 – 3 d)**	15 (2)	31 (4)	.02
**Urgent (to OR within 2 – 24 h)**	63 (8)	93 (12)	.01
**Mean procedure duration (min)**	171	181	.06
**Outcomes**			
**AP infusions initiated and completed correctly within the specified preincision window**			
**MTZ (120 min) Cefazolin (60 min)**	4/671 (1) 685/726 (94)	332/671 (49) 688/719 (96)	< .0001 .28
**Median time of initiation prior to incision (min)**			
**MTZ (30 min infusion)** **Cefazolin (3 – 5 min push)**	13 (IQR: 6 – 23) 17 (IQR: 10 – 27)	98 (IQR: 56 – 131) 17 (11 – 26)	
**SSI, *n* (incidence rate)**	35 (46.4/100,000)	38 (50.9/100,000)	.72
**Type of infusion timing error for MTZ:**			
**Infusion initiated too late**	660 (98)	128 (19)	< .0001
**Infusion initiated too early**	7 (1)	211 (32)	< .0001

MTZ, metronidazole; SSI, surgical site infection. Data are reported as *n* (%), unless otherwise specified.

## Discussion

This quality improvement project highlights the importance of paying attention to infusion timing for perioperative antibiotics that are not β-lactams, using MTZ as a case example. Unlike cefazolin, which can be administered rapidly, MTZ requires a longer infusion. Evidence suggests that administering antimicrobials 30 – 120 min before incision is more effective than the previously recommended 15 – 30 min window. When administered in the operating room, infusions were often incomplete at the time of incision, potentially reducing prophylactic effectiveness.^8^

Optimizing perioperative antimicrobial administration can be challenging and needs to be recognized as a patient priority to get multidisciplinary team engagement to overcome workflow barriers.^9^ Waiting for completion in the operating room was viewed as impractical due to concerns about workflow and case delays. Not only did this process change improve adherence to prophylaxis principles, it also engaged a broader group of providers in stewardship practices and underscored the importance of considering antibiotic infusion characteristics in perioperative planning. Additionally, the intervention promoted alignment across surgical services to adopt standardized order sets, allowing for better anticipation and delivery of appropriate antibiotics before surgery.

Although no statistically significant reduction in SSI was observed, interpretation is limited by the sample size and a higher proportion of factors associated with increased SSI risk (e.g., urgent cases and older patients) in the postintervention group.^8^ Importantly, cefazolin administration timing remained unchanged, underscoring that the effect was specific to MTZ and the process change of shifting the administration to a different group of providers and increasing the preincision time frame for initiation to 120 min. While there was a sharp increase in the proportion of MTZ being given too early (as opposed to frequently too late in the preintervention group), its pharmacodynamics and longer half-life favor an earlier rather than later administration time-similar to vancomycin.^2^ In summary, recognizing infusion time differences in dual-agent prophylaxis and establishing different practitioners in giving different AP to ensure completion before surgery may be essential to maximize the effectiveness of perioperative antimicrobials.
